# Foreign-born people and covid-19 in Sweden - the effect of co-morbidity: Sharareh Akhavavn

**DOI:** 10.1093/eurpub/ckac129.732

**Published:** 2022-10-25

**Authors:** S Akhavavn

**Affiliations:** National Board of Health and Welfare, Stockholm, Sweden

## Abstract

Foreign-born people who live in Sweden, especially those who were born in low- and middle-income countries, were at higher risk of developing severe covid-19. It has previously been found that morbidity and mortality in covid-19 were associated with country of birth, level of education, income and type of housing. The aim of this study was to map differences between foreign and Swedish-born (18 years and older) with severe covid-19 regarding co-morbidity. This was a register -base study. Register data were gathered from the Patient Register, the Swedish Intensive Care Register, the Cause of Death Register and information on socio-economic variables from Statistics Sweden. Results shows that co-morbidity with severe covid-19 was generally higher for Swedish-born than foreign-born. On the other hand, co-morbidity with diabe-tes and obesity was slightly higher for foreign-born than for Swedish-born. The results also indicate that a higher risk of developing severe covid-19 remains despite the fact that we have controlled a number of factors that affect the relationships such as residential region, demography and socioeconomics. The conclusion of this study is that there is a need for strengthening public health surveillance systems for information such as diagnoses from primary care, the stage at which a person who has become infected has sought care, how the possible differences were in care seekers between foreign and Swedish-born and what the care chain looked like from the time the patient received first symptoms until she/he ended up in inpatient or intensive care.

**Key messages:**

• Co-morbidity with severe covid-19 was generally higher for Swedish-born than foreign-born.

• There is a need for strengthening public health surveillance systems in primary health care for explaining the reasons of higher risk of developing severe covid-19 among foreign-born people in Sweden.

